# *Micrographia* of the twenty-first century: from *camera obscura* to 4D microscopy

**DOI:** 10.1098/rsta.2009.0265

**Published:** 2010-03-13

**Authors:** Ahmed H. Zewail

**Affiliations:** Physical Biology Center for Ultrafast Science and Technology, Arthur Amos Noyes Laboratory of Chemical Physics, California Institute of Technology, Pasadena, CA 91125, USA

**Keywords:** light, electrons, microscopy

## Abstract

In this paper, the evolutionary and revolutionary developments of microscopic imaging are overviewed with a perspective on origins. From Alhazen’s *camera obscura*, to Hooke and van Leeuwenhoek’s two-dimensional optical micrography, and on to three- and four-dimensional (4D) electron microscopy, these developments over a millennium have transformed humans’ scope of visualization. The changes in the length and time scales involved are unimaginable, beginning with the visible shadows of candles at the centimetre and second scales, and ending with invisible atoms with space and time dimensions of sub-nanometre and femtosecond. With these advances it has become possible to determine the structures of matter and to observe their elementary dynamics as they unfold in real time. Such observations provide the means for visualizing materials behaviour and biological function, with the aim of understanding emergent phenomena in complex systems.

## 1. Origins in light

**Figure 1.**
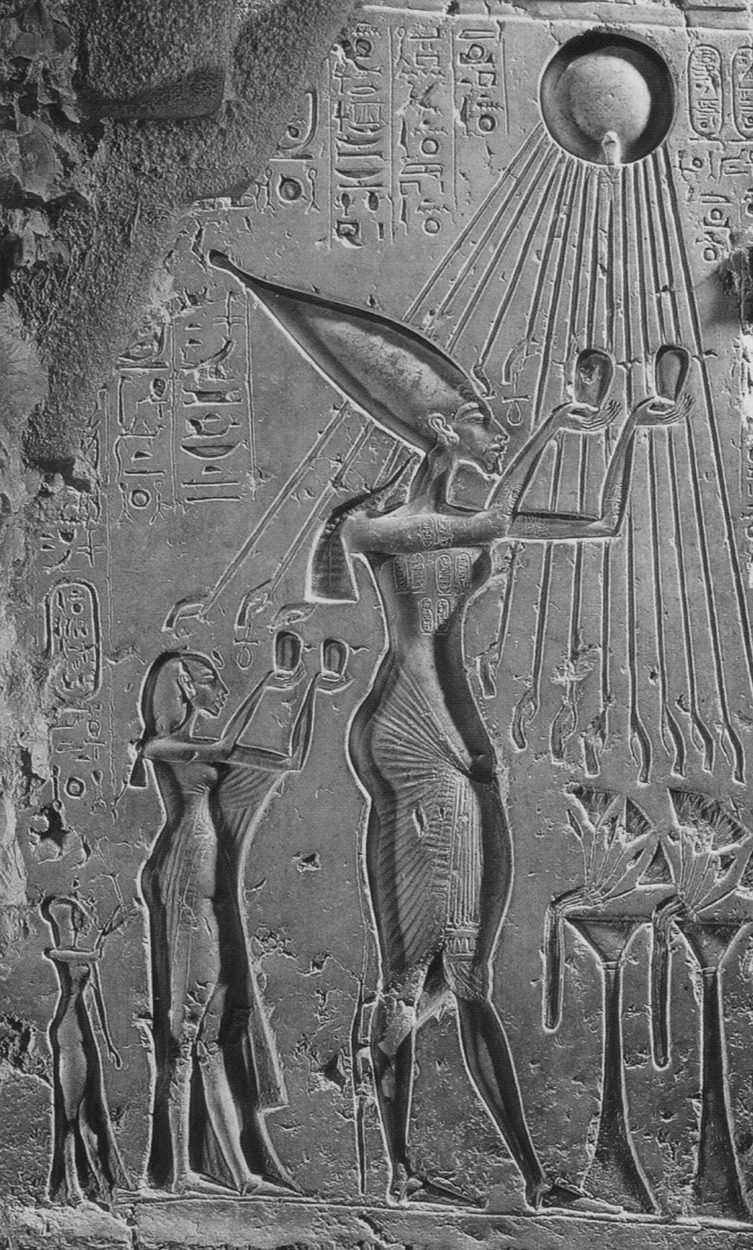
The significance of the light–life interaction as perceived more than three millennia ago, at the time of Akhenaton and Nefertiti. Note the light’s ‘ray diagram’ from a spherical source, the Sun. Adapted from Zewail (2008).

The ever-increasing progress made by humans in making the very small and the very large visible and tangible is truly remarkable. The human eye is not diffraction limited, but its spatial and temporal resolutions are limited to about 100 μm and a fraction of a second, respectively. Today we are aided by tools that enable the visualization of objects that are below a nanometre in size and that move in femtoseconds or attoseconds (Zewail & Thomas 2009, and references therein). How did it all begin? Surely the power of light for observation has been with humans since their creation. Stretching back over six millennia, one finds its connection to the science of time clocking (Zewail 2000) (first in calendars) and to the mighty monotheistic faiths and rituals (figure 1).

**Figure 2.**
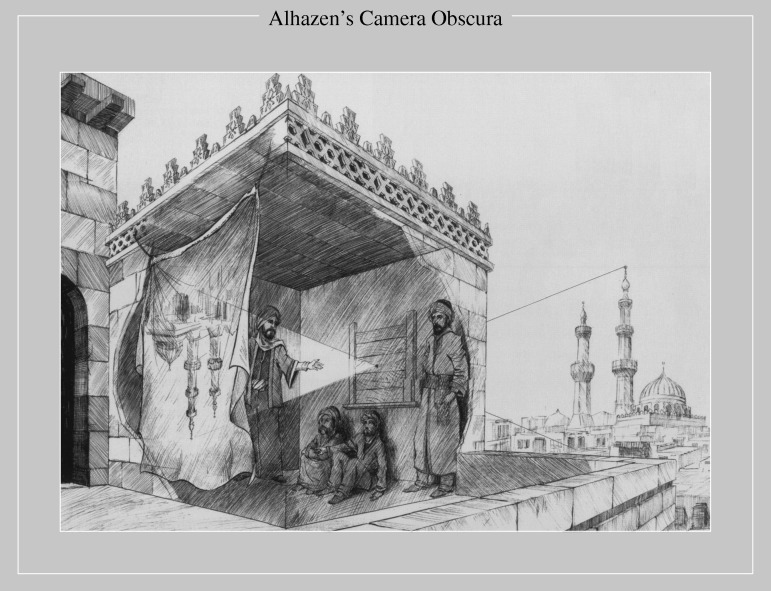
The concept of the *camera obscura* as perceived a thousand years ago by Alhazen (Ibn al-Haytham), who coined the term (see text). Note the formation of the inverted image through a ray diagram. Adapted from Al-Hassani *et al.* (2006).

Naturally, the philosophers of the past must have been baffled by the question: what is light and what gives rise to the associated optical phenomena? A leading contribution to this endeavour was made by the Arab polymath Alhazen (Ibn al-Haytham; AD 965–1040). He is recognized for his quantitative experimentation and thoughts on reflection and refraction, and is also credited with correctly explaining the mechanism of vision, prior to the contributions of Kepler, Descartes, Da Vinci, Snell and Newton. But of relevance to our topic is his conceptual analysis of the *camera obscura*, the ‘dark chamber’, which aroused the photographic interests of J. W. Strutt (later known as Lord Rayleigh) in the 1890s (Strutt 1891). Alhazen’s idea that light must travel along straight lines and that the object is inverted in the image plane is no different from the modern picture of ray diagrams taught in optics today (figure 2). His brilliant work was published in the *Book of Optics* or, in Arabic, *Kitab al-Manazir*.

**Figure 3.**
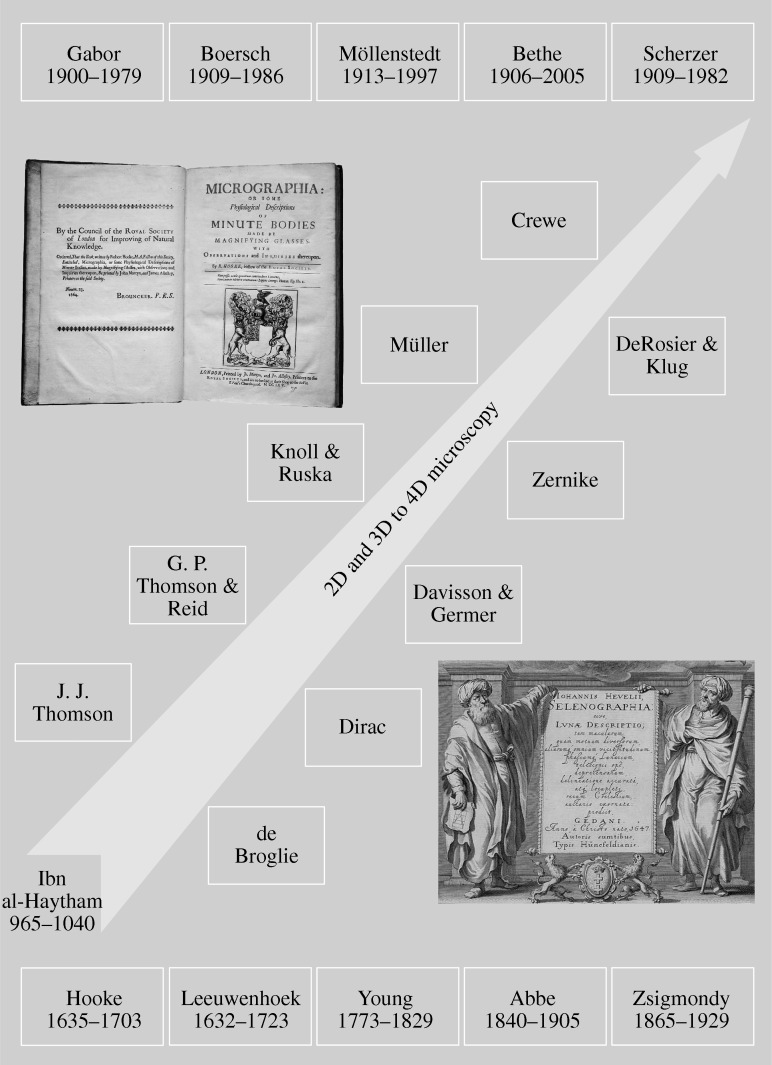
Microscopy time line, from *camera obscura* to three-dimensional electron microscopes. 4D ultrafast electron microscopy and diffraction were developed a decade ago (see text). The top inset shows the frontispiece to Hooke’s (1665)*Micrographia* published by the Royal Society of London. In the frontispiece to Hevelius’s *Selenographia* (bottom inset), Ibn al-Haytham represents *Ratione* (the use of reason) with his geometrical proof and Galileo represents *Sensu* (the use of the senses) with his telescope. The two scientists hold the book’s title page between them, suggesting a harmony between the methods (Sabra 2003; Steffens 2006; Zewail & Thomas 2009).

In the fourteenth and fifteenth centuries, the art of grinding lenses was perfected in Europe, and the idea of optical microscopy was developed. In 1665, Robert Hooke (the man who coined the word ‘cell’) published his studies in *Micrographia* (Hooke 1665; figure 3), and among them was a description of plants, feathers, as well as cork and its ability to float in water. Contemporaneously, Anton van Leeuwenhoek used a simple, one-lens microscope to examine blood, insects and other objects, and was the first to visualize bacteria, among other microscopic objects. More than a hundred years later, an experiment by the physicist, physician and Egyptologist, Thomas Young, demonstrated the interference of light, an experiment that revolutionized our views on the nature of light. His double-slit experiment of 1801 performed at the Royal Institution of Great Britain led to the demise of Newton’s corpuscular theory of light. Of relevance here is the phenomenon of diffraction due to interferences of waves (coherence). Much later, such diffraction was found to yield the (microscopic) interatomic distances characteristic of molecular and crystal structures, as discovered in 1912 by von Laue and elucidated later that year by W. L. Bragg.

Resolution in microscopic imaging was brought to a whole new level by two major developments in optical microscopy. In 1878, Ernst Abbe formulated a mathematical theory correlating resolution to the wavelength of light (beyond what we now designate the empirical Rayleigh criterion for incoherent sources), and hence the optimum parameters for achieving higher resolution. At the beginning of the twentieth century, Richard Zsigmondy, by extending the work of Faraday and Tyndall, developed the ‘ultramicroscope’ to study colloidal particles; for this work, he received the Nobel Prize in Chemistry in 1925. Then came the penetrating developments in the 1930s by Frits Zernike, who introduced the phase-contrast concept in optical microscopy; he, too, received the Nobel Prize, in Physics, in 1953. It was understood that the spatial resolution of optical microscopes was limited by the wavelength of the visible light used. Recently, optical techniques have led to considerable improvement in spatial resolution, as discussed below.

## 2. Electrons in microscopy

Just before the dawn of the twentieth century, in 1897, electrons, or the *corpuscles* of J. J. Thomson, were discovered, but they were not conceived as imaging rays until Louis de Broglie formulated the concept of particle–wave duality in 1924. The duality character of an electron, which is quantified in the relationship *λ*_de Broglie_=*h*/*p*, suggested the possibility of achieving waves of picometre wavelength, and became essential to the understanding of diffraction and imaging. The first experimental evidence of the wave character of the electron was established in 1927 by Davisson and Germer (diffraction from a nickel surface) and, independently, by G. P. Thomson (the son of J. J. Thomson), who, with Reid, observed diffraction of electrons penetrating a thin foil. In 1923, Dirac postulated the concept of ‘single-particle interference’.

Later, Knoll & Ruska (1932) invented the electron microscope (EM) and improved the resolution to the (sub)micrometre scale in the transmission electron microscope (TEM). Boersch introduced the diffraction lens in TEM in 1936, and later (1940) he found the so-called Fresnel fringes as ‘diffraction at edges’ in the microscope. Concurrently, Walther Kossel and Gottfried Möllenstedt in 1939 combined in their EM the ability to record projected two-dimensional images and electron diffraction patterns, which contain information on the structure, the repeating lattice distances and other aspects pertaining to crystallographic symmetry. These and other related developments in microscopy led to electron interferometry and holography. The original proposal of electron holography by Denis Gabor in 1948 and the birth of electron biprism interference by Möllenstedt in 1953 laid the foundation (Silverman *et al.* 1995; Lichte 2002, and references therein; Spence 2009) for the impressive advances made by Tonomura (1998, 1999) and others in the years to follow.

## 3. Imaging atoms, molecules and cells

The first images of individual atoms were obtained in 1951 by Müller (Müller 1951; Tsong 2006; Thomas 2008), who introduced the technique of field-ion microscopy to visualize them at fine tips of metals and alloys, and to detect vacancies and atomic steps and kinks at the surfaces. With the invention of field-emission sources and scanning TEM, pioneered in 1970 by Crewe, isolated heavy atoms became readily visible (Crewe *et al.* 1970; Thomas 1979). (The scanning tunnelling microscope was developed in the 1980s and made possible atomic-scale images of conducting surfaces.) Today, with aberration-corrected microscopes, imaging has reached a resolution of less than an ångström (Nellist *et al.* 2004). This history would be incomplete if I did not mention that the totality of technical developments and applications in the investigations of inorganic and organic materials have benefited enormously from the contributions of many other scientists, and for more details I refer the reader to the books by Cowley (1995), Humphreys (2002), Gai & Boyes (2003), Spence (2003) and Hawkes & Spence (2007), and the most recent papers by Hawkes (2009) and Howie (2009).

Biological EM has been transformed by several major advances, including electron crystallography, single-particle tomography and cryo-microscopy, aided by large-scale computational processing. Beginning with the 1968 electron crystallography work of DeRosier and Klug (see Klug 1982), three-dimensional density maps became retrievable from EM images. Landmark experiments revealing the high-resolution (atomic-scale) structure from two-dimensional crystals, single-particle three-dimensional cryo-EM images of different but identical particles (6 Åresolution) and three-dimensional cryo-EM images of the same particle (tomography with 6 Å resolution) represent the impressive progress made. With these methods, the first membrane protein structure was determined, the first high-resolution density maps for the protein shell of an icosahedral virus were obtained, and the imaging of whole cells was accomplished. Minimizing radiation damage by embedding the biological macromolecules and machines in vitreous ice affords a non-invasive, high-resolution imaging technique for visualizing the three-dimensional organization of eukaryotic cells, with their dynamic organelles, cytoskeletal structure and molecular machines in an unperturbed context, with a resolution of 6 Å to 2 nm, being limited by radiation damage. I refer the reader to the papers by Henderson (1995), Sali *et al.* (2003), Crowther (2008) and Glaeser (2008), and the books by Glaeser *et al.* (2007) and by Frank (2006).

## 4. 4D electron microscopy

**Figure 4.**
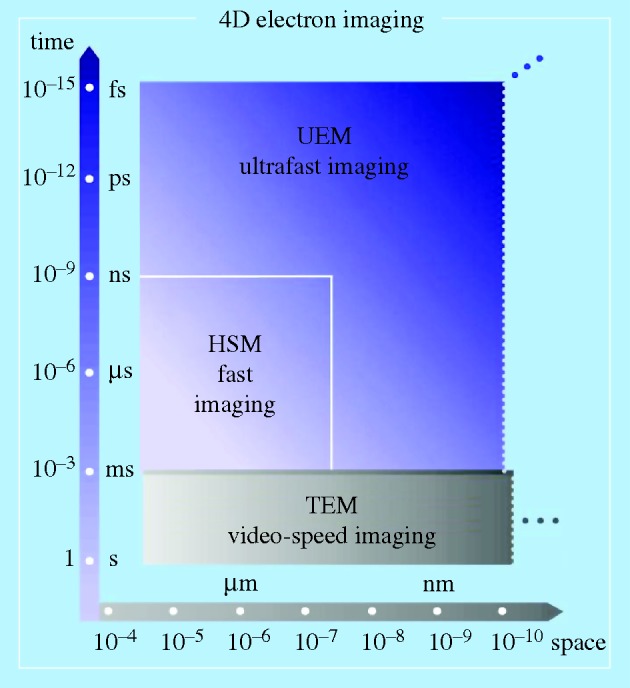
Resolutions in space and time achieved in electron microscopy. The focus here is on the comparison of ultrafast electron microscopy (UEM) and transmission electron microscopy (TEM), but other variants of the techniques (scanning EM, tomography and holography, as well as electron spectroscopy) can similarly be considered. The horizontal dimension represents the spatial resolution achieved from the early years of EM to the era of aberration-corrected instruments. The vertical axis depicts the temporal resolution scale achieved up to the present time and the projected extensions into the near future. The domains of ‘fast’ and ‘ultrafast’ temporal resolutions are indicated by the areas of high-speed microscopy (HSM) and ultrafast electron microscopy (UEM) (Zewail & Thomas 2009). Vertical dotted lines separate the spatial resolutions characteristic of real-space (microscopy) imaging from the spatial resolutions that are obtainable using the reciprocal-space (diffraction) techniques, which reach the picometre scale.

**Figure 5.**
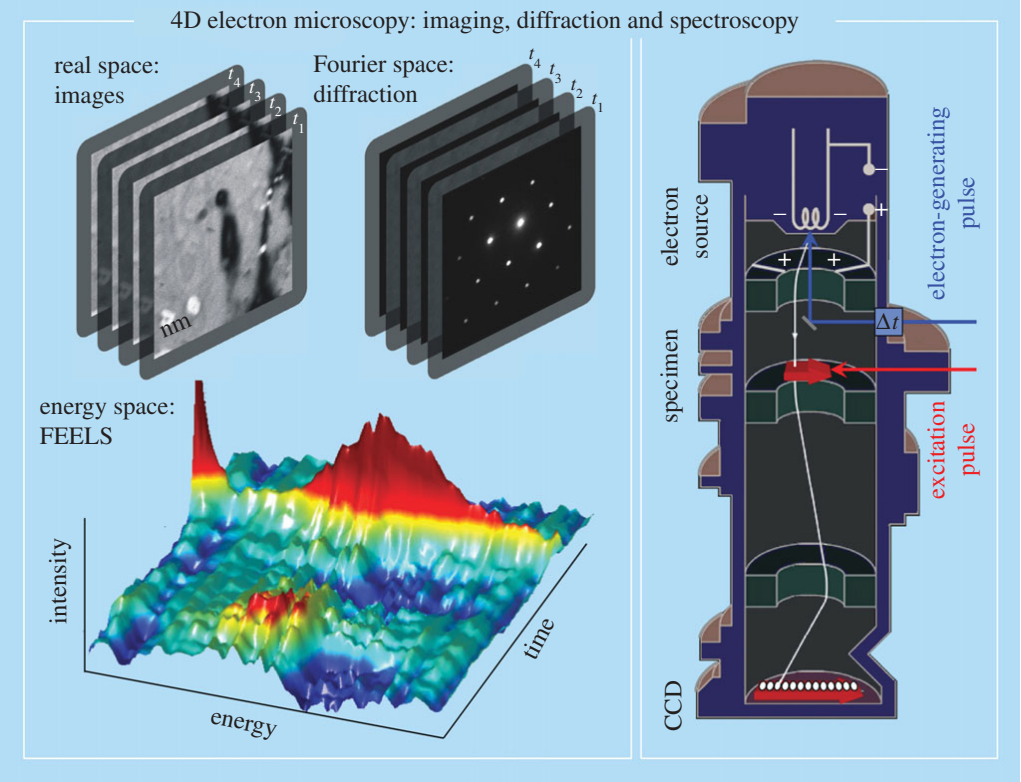
4D electron imaging in real, Fourier and energy spaces. The conceptual design of Caltech’s UEM-2 is presented on the right; a single-electron trajectory is depicted within the UEM. The atomic-scale (femtosecond) temporal resolution characteristic of the apparatus allows for the visualization of dynamical processes in real time. Shown on the left are typical UEM frames of real-space images and diffraction patterns, together with three-dimensional maps of femtosecond-resolved electron-energy-loss spectra (FEELS). For a recent review, see Shorokhov & Zewail (2009).

Whereas in all of the above methods the processes of imaging, diffraction and chemical analysis have been conducted in a *static* (time-averaged) manner, it has now become possible to unite the time domain with the spatial one, thereby creating 4D electron microscopy (Barwick *et al.* 2008; Carbone *et al.* 2009; Yurtsever & Zewail 2009; Barwick *et al.* 2009); for a recent review, see Shorokhov & Zewail (2009). This development owes its success to the advancement of the concept of coherent *single-electron imaging*, with the electron packets being liberated from a photocathode using femtosecond optical pulses. In such a mode of electron imaging, the repulsion between electrons is negligible, and thus atomic-scale spatiotemporal resolution can be achieved. Atomic motions, phase transitions, mechanical movements and the nature of fields at interfaces are examples of phenomena that can be charted in unprecedented structural detail at a rate that is ten orders of magnitude faster than hitherto. Furthermore, because electrons are focusable and can be pulsed at these very high rates, and because they have appreciable inelastic cross sections, the EM yields information in four distinct ways: in real space, in reciprocal space, in energy space and in the time domain. Convergent beam imaging was also shown to provide nanoscale diffraction of heterogeneous structures (Yurtsever & Zewail 2009), and near-field imaging can map nanoscale electromagnetic fields of material structures (Barwick *et al.* 2009). Thus, besides structural imaging, the energy landscapes of macromolecules may be explored; and, under optimal conditions, elemental compositions, valence-states bonding and three-dimensional information (from tomography) may also be retrieved.

 figure 4 depicts the space and time dimensions of TEM and of ultrafast electron microscopy (UEM). The boundaries of the time resolution are representative of the transition from the millisecond video speed used in TEM imaging, to fast or high-speed (nanosecond to microsecond) imaging, and on to the ultrafast (femtosecond to picosecond) imaging regime. The spatial resolution in the high-speed, nanosecond domain indicated in the figure is limited by electron–electron (space–charge) repulsion in the nanosecond pulses of electrons. The UEM landscape is that of single-electron imaging, which, owing to the absence of inter-electron repulsion, reaches the spatial resolution of the TEM. Examples of time-averaged EM and of UEM studies can be found in Zewail & Thomas (2009, and references therein). The key concepts involved in the UEM and some prototypical results are provided in figure 5.

The concept of single-electron imaging is based on the premise that the trajectories of coherent and timed, single-electron packets can provide an image equivalent to that obtained using many electrons in conventional microscopes. Unlike the random electron distribution of conventional microscopes, in UEM the packets are timed with femtosecond precision, and each electron has a unique coherence volume. As such, each electron of finite de Broglie wavelength is (transversely) coherent over the object length scale to be imaged, with a longitudinal coherence length that depends on its velocity. On the detector, the electron produces a ‘click’ behaving as a classical particle, and when a sufficient number of such clicks is accumulated stroboscopically, the whole image emerges. Putting it in Dirac’s famous dictum: *each electron interferes only with itself*. In the microscope, the electron pulse that produces the image is termed the *probe* pulse, and in ultrafast imaging with a train of such pulses, the number of frames in a movie could then be as high as 10^12^ per second; this ‘stop-motion photography’ constitutes a real-time movie of the process.

To visualize the motion, the molecule or material must be launched on its path using a femtosecond initiation pulse, the *clocking* or *pump* pulse, thus establishing a temporal reference point (time zero) for the changes that occur in the motion. By sending the clocking pulse along an adjustable optical path, we can precisely fix each probe frame on the time axis—knowing the speed of light, a typical optical path accuracy of 1 μm corresponds to absolute timing of the snapshots of 3.3 fs. Because the clocking pulse is controlled to precede each electron pulse, the time axis is defined by the separation between them and is no longer limited by the response of the video detector in the microscope. Lastly, in order to synchronize the motion of many independent atoms or molecules so that all of them have reached a similar point in the course of their structural evolution, the relative timing of clocking and probe pulses must be of femtosecond precision, and the launch configuration must be defined to sub-ångström resolution.

In imaging with electrons, unlike with photons, we must also consider the consequences of the Pauli exclusion principle. The maximum number of electrons that can be packed into a state (or a cell of phase space) is two, one for each spin; in contrast, billions of photons can be condensed in a state of the laser radiation. This characteristic of electrons represents a fundamental difference in what is termed the ‘degeneracy’, or the mean number of electrons per cell in phase space. Typically it is about 10^−4^–10^−6^ but it is possible in UEM to increase the degeneracy by orders of magnitude, a feature that could be exploited for studies in quantum electron optics (Zewail & Thomas 2009). I note here that the definition of ‘single-electron packet’ is reserved for the case when each timed packet contains one or a small number of electrons such that coulombic repulsion is effectively absent.

At Caltech, two UEM microscopes operate at 120 and 200 keV. Upon the initiation of the structural change by heating of the specimen, or through electronic excitation, by the ultrashort clocking pulse, a series of frames for real-space images, and similarly for diffraction patterns or electron-energy-loss spectra (EELS), is obtained. In the single-electron mode of operation, which affords studies of reversible processes or repeatable exposures, the train of strobing electron pulses is used to build up the image. By contrast, in the single-pulse mode, each recorded frame is created with a single pulse that contains 10^5^–10^6^ electrons. One has the freedom to operate the apparatus in either single-electron or single-pulse mode.

Shortly after the first EM was built by Knoll and Ruska, it was realized that the resolution of the instrument, working under ideal conditions, would far exceed that attainable using a light microscope. It is known from the Rayleigh criterion that, with a wavelength of *λ* for the probing beam, the smallest distance that can be resolved is given by approximately 0.5*λ*. Thus, in conventional optical microscopy, green light cannot resolve distances smaller than approximately 3000 Å(300 nm). Special elegant variants of optical microscopy can nowadays resolve small objects of several tens of nanometres, below the diffraction limit (Klar *et al.* 2000; Freudiger *et al.* 2008; Huang *et al.* 2009). When, however, an electron is accelerated in a 100 kV microscope, its wavelength approaches 4×10^−2^ Å, reaching the picometre scale, a distance far shorter than that separating the atoms in a solid or molecule. Equally important, electron imaging provides structural information (shapes), whereas light microscopes, with the best resolution, and using fluorophores, provide only positions (coordinates). For a variety of reasons, a principal one being the inherent imperfection of electron-optical lenses, it is not yet possible to achieve the theoretical limit of resolution set by the wavelength of the electron. However, steady advances have been made, especially in recent years, in achieving resolutions of less than 1 Å, due to the arrival of so-called aberration-corrected electron lenses.

Of the three kinds of primary beams (neutrons, X-rays and electrons) suitable for structural imaging, the most powerful are coherent electrons, which are readily produced from field emission guns. The source brightness, as well as the temporal and spatial coherence of such electrons, significantly exceeds the values achievable for neutrons and X-rays: moreover, the minimum probe diameter of an electron beam is as small as 1 Å, and its elastic mean free path is approximately 100 Å (for carbon), much less than for neutrons and X-rays. For larger samples and for those studied in liquids, X-ray absorption spectroscopy and diffraction, when time-resolved, provide unprecedented details of energy pathways and molecular structural changes (Bressler *et al.* 2009; Chergui & Zewail 2009; Kim *et al.* 2009). It is significant to note that in large samples the precision is high but it represents an average over micrometre-scale specimens. When electron microscopy is invoked, the high resolution in real space can reveal defects of structures at the atomic scale. Such defects were shown to be critical in Gai’s seminal studies of catalysis by environmental TEM (Gai & Boyes 2003), and they are local in nature.

As a result of these developments and inventions, new fields of research are now emerging. First, by combining energy-filtered electron imaging with electron tomography, chemical compositions of sub-attogram (less than 10^−18^ g) quantities located at the interior of microscopic or mesoscopic objects may be retrieved non-destructively. Second, transmission electron microscopes fitted with field-emission guns to provide coherent electron waves can be readily adapted for electron holography to record the magnetic fields within and surrounding nanoparticles or metal clusters, thereby yielding the lines of force of, for example, a nanoferromagnet encapsulated within a multi-walled carbon nanotube. Third, advances in the design of aberration-corrected high-resolution EMs have greatly enhanced the quality of structural information pertaining to nanoparticle metals, binary semiconductors, ceramics and complex oxides. Moreover, electron tomography sheds light on the shape, size and composition of materials. Finally, with convergent-beam and near-field 4D UEM (Yurtsever & Zewail 2009; Barwick *et al.* 2009), the structural dynamics of a nanoscale single site (particle), and of nanoscale interface fields, can be visualized, reaching the atomic scale and beyond (Zewail & Thomas 2009).

## 5. Visualization and complexity

Realization of the importance of visualization and observation is evident in the exploration of natural phenomena, from the very small to the very large. A century ago, the atom appeared complex, a ‘raisin or plum pie of no structure’, until it was visualized on the appropriate length and time scales. Similarly, with telescopic observations, a central dogma of the cosmos was changed and complexity yielded to the simplicity of the heliocentric structure and motion in the entire Solar System. From the atom to the Universe, the length and time scales span extremes of powers of 10. The electron in the first orbital of a hydrogen atom has a ‘period’ of sub-femtoseconds, and the size of atoms is on the nanometre scale or less. The lifetime of our Universe is approximately 13 billion years and, considering the light year (approx. 10^16^ m), its length scale is of the order of 10^26^ m. In between these scales lies the world of life processes, with scales varying from nanometres to centimetres and from femtoseconds to seconds.

In the early days of DNA structural determination (1950s), a cardinal concept, in vogue at that time, was encapsulated in Francis Crick’s statement: *If you want to know the function, determine the structure*. This view pervaded the thinking at the time, and it was what drove Max Perutz and John Kendrew earlier in their studies of proteins. But as we learn more about complexity, it becomes clear that the so-called ‘structure–function’ correlation is insufficient to establish the mechanisms that determine the behaviour of complex systems. For example, the structures of many proteins have been determined, but we still do not understand how they fold, how they selectively recognize other molecules, how the matrix water assists folding and the role it plays in directionality, selectivity and recognition. The proteins haemoglobin and myoglobin (a subunit of haemoglobin) have unique functions: the former is responsible for transporting oxygen in the blood of vertebrates, while the latter carries and stores oxygen in muscle cells. The three-dimensional structures of the two proteins have been determined (by Perutz and Kendrew), but we still do not understand the differences in behaviour in the oxygen uptake by these two related proteins, the role of hydration, and the exact nature of the forces that control the dynamics of oxygen binding and liberation from the haem group. Visualization of the changing structures during the course of their functional operation is what is needed.

A supreme example of large-scale complexity is evident in correlated physical systems exhibiting phase transitions or self-assembly, and in biological systems with emergent behaviour (Zewail 2008). For materials, an assembly of atoms in a lattice can undergo a change, which leads to a new structure with properties different from the original ones. In other materials, the structural transformation leads to a whole new material phase, as in the case of metal–insulator phase transitions. Questions of fundamental importance pertain to the time and length scales involved and to the elementary pathways that describe the mechanism. Recently, a number of such questions have been addressed by means of 4D electron imaging. Of significance are two regimes of structural transformation: the first involves an initial (coherent) bond dilation that triggers unit-cell expansion and phase growth (Baum *et al.* 2007), and the second involves phase transformations in a diffusionless (collective) process that emerges from an initial random motion of atoms (Park *et al.* 2009).

In biological transformations, the energy landscape involves very complicated pathways, including those that lead to a multitude of conformations, with some that are ‘active’ and others that are ‘inactive’ in the biological function. Moreover, the landscapes define ‘good’ and ‘bad’ regions, the latter being descriptive of the origin of molecular diseases. It is remarkable that the robustness and function of these ‘molecules of life’ are the result of a balance of weak forces—hydrogen bonding, electrostatic forces, dispersion and hydrophobic interactions—all of energy of the order of a few kcal mol^−1^, or approximately 0.1 eV or less. Determination of time-averaged molecular structures is important and has led to an impressive list of achievements, for which more than ten Nobel Prizes have been awarded, but the structures relevant to function are those that exist in the non-equilibrium state. Understanding their behaviour requires an integration of the trilogy: structure, dynamics and function. Experimental and theoretical efforts (Lin *et al.* 2006, 2008, 2009*a*,*b*; Zewail & Thomas 2009, and references therein) have been launched to explore these areas of research pertaining to biological structures and energy landscapes.

## 6. Epilogue

The microscope is arguably one of the two most powerful human-made instruments of all time, the other being the telescope. To our vision they brought the very small and the very far. Robert Hooke, for his *Micrographia*, chose the subtitle: *or some physiological descriptions of minute bodies made by magnifying glasses with observations and inquiries thereupon*. These words were made in reference to conventional optical microscopes, the spatial resolution of which is limited by the wavelength of visible light, the Rayleigh criterion. The transmission electron microscope, since its invention in the 1930s, has provided the wavelength of picometres, taking the field of imaging beyond the ‘minutes’ of the seventeenth century *Micrographia*—it has now become possible to image individual atoms, and the scope of applications spans essentially all of the physical sciences as well as biology. With 4D microscopy, the structures determined are no longer time-averaged over seconds of recording. They can be seen as frames of a movie that elucidates the nature of the processes involved. We have come a long way from the epochs of the *camera obscura* and Hooke’s *Micrographia*, but I am confident that new research frontiers will continue to emerge in the twenty-first century, especially at the intersection of physical, chemical and biological sciences (Zewail 2009).
